# Gender Differences in the Association Between Cataract and Mental Health in Adults With Diabetes: A Cross-Sectional Analysis From the Spanish National Health Survey 2017

**DOI:** 10.3389/fpubh.2021.769155

**Published:** 2021-12-07

**Authors:** Guillermo F. López Sánchez, Lee Smith, Louis Jacob, Jae Il Shin, Ai Koyanagi, Shahina Pardhan

**Affiliations:** ^1^Faculty of Health, Education, Medicine and Social Care, School of Medicine, Vision and Eye Research Institute, Anglia Ruskin University, Cambridge, United Kingdom; ^2^The Cambridge Centre for Sport and Exercise Sciences, Anglia Ruskin University, Cambridge, United Kingdom; ^3^Research and Development Unit, Parc Sanitari Sant Joan de Déu, Centro de Investigación Biomédica en Red de Salud Mental (CIBERSAM), Sant Boi de Llobregat, Barcelona, Spain; ^4^Faculty of Medicine, University of Versailles Saint-Quentin-en-Yvelines, Montigny-le-Bretonneux, France; ^5^Department of Pediatrics, Yonsei University College of Medicine, Seoul, South Korea; ^6^Institució Catalana de Recerca i Estudis Avançats (ICREA), Barcelona, Spain

**Keywords:** cataracts, chronic anxiety, depression, diabetes, gender

## Abstract

**Objective:** This study aimed to explore gender differences in the associations between cataracts and self-reported depression and chronic anxiety in Spanish adults with diabetes.

**Methods:** Cross-sectional data from the Spanish Health Survey 2017 were analyzed. Inclusion criterion was a positive response to the question “Have you ever been diagnosed with diabetes?” Diabetes, cataracts, depression and chronic anxiety were based on self-reported lifetime diagnosis. Multivariable logistic regression was conducted to assess the association between cataracts and depression or anxiety among respondents with diabetes, stratifying by gender.

**Results:** Out of a total 23,089 respondents, 2,266 people self-reported suffering from diabetes (50.2% women; average age 69.7 ± 12.7 years; age range 15-98 years). In people with diabetes, the presence of cataracts was associated with significantly higher odds for depression (OR = 1.655; 95% CI = 1.295-2.115). Gender-stratified analyses showed that only women with cataracts were significantly associated with higher odds for depression (OR = 1.762; 95% CI = 1.307-2.374) and chronic anxiety (OR = 1.519; 95% CI = 1.067-2.163).

**Conclusion:** Cataracts are a significant risk factor for depression and chronic anxiety in Spanish women with diabetes, but not in men. Women with both diabetes and cataracts require assessment for depression and chronic anxiety, and possibly earlier interventions in order to reduce the potential risk of further mental health complications.

## Introduction

Globally ~422 million people live with diabetes and prevalence of the condition has been increasing steadily over recent decades (1). In Europe, there are approximately 60 million people with the condition, about 10.3% of men and 9.6% of women aged 25 years and over (2). In Spain, the incidence of diabetes, adjusted for age and gender has been reported to be 11.6 cases/1,000 person-years (95%CI = 11.1-12.1) (3).

Diabetes is associated with multiple health complications. One complication is visual impairment with cataract being a key cause (4). Indeed, people with diabetes are up to five times more likely to develop cataracts compared to those without diabetes (5, 6). Both visual impairment and diabetes separately have been linked to higher levels of depression and anxiety than in the general population. For example, one meta-analysis of seven studies concluded that diabetes is associated with an increased risk of depression (7), and another showed significant positive associations with anxiety disorders (1.20; 95% CI = 1.10-1.31) and elevated anxiety symptoms (1.48; 95%CI = 1.02-1.93), with a pooled OR for all anxiety studies (1.25; 95% CI = 1.10-1.39) (8). Furthermore, another systematic review (9) and a 16-year nationwide population-based longitudinal study (10) suggested that a strong link exists between cataracts and depression. Higher levels of anxiety in people with cataracts have also been suggested (11).

Bener et al. reported that significantly more women with diabetes had depression (63.3 vs. 50.4%) and anxiety (70.1 vs. 61.6%) compared to men (12). With regards to the prevalence of depression in people with cataracts by gender, Liu et al. (13) found that there was a similar prevalence of depression in women (24.2%) and men (23.6%).

Given that diabetes and cataracts are both associated with higher risk for mental health problems, it is possible that people with co-existing diabetes and cataracts would have higher risk of depression and anxiety. In addition, although gender differences have been previously found in prevalence studies for depression and anxiety, it is not known whether gender differences exist in mental health in people who have both diabetes and cataract. Therefore, in accordance with the international recommendations about gender in public health research (14, 15), the aim of the present study was to explore gender differences in the associations between cataracts and self-reported depression and chronic anxiety in Spanish adults with diabetes.

## Methods

### The Survey

Data from the Spanish National Health Survey 2017 (*n* = 23,089 adults) were analyzed. This survey was carried out in Spain between October 2016 and October 2017. Details of the survey methods have been published elsewhere (16). A stratified three-stage sampling technique was used to collect the data, considering first census sections, second family dwellings, and third an adult (15 years or more) in each dwelling. The selection of each section within each stratum was carried out with probability proportional to their size. In each section, the dwellings were selected by systematic sampling with equal probability, prior arrangement by dwelling size. This method produces self-weighting samples in each stratum. The random Kish method was used to select the people who had to complete the Adult Questionnaire, assigning equal probability to all the adults of the household. The inclusion criterion to participate in this study was a positive response the question “Have you ever been diagnosed with diabetes?” Therefore, the sample of the present study included the 2,266 people with diabetes of the survey (9.8% of the total survey sample), being representative of the adult population with diabetes residing in Spain. Also, this prevalence of 9.8% is in line with previous studies in Spain, which reported prevalence's from 4.8 to 18.7% (17).

### Procedure

The method of data collection used was computer-assisted personal interviewing (CAPI), conducted in the homes of the selected participants. The interviewers, previously trained, completed the questionnaires with the information provided by the participants. The interviews were carried out in the selected dwellings. The personnel in charge of conducting the interviews were assigned a periodic work quota distributed according to the sample design. The interviewer had to make at least 6 visits on three different days to the dwelling, until contacting the home or giving the corresponding incidence. The field work (data collection, inspection, monitoring and first data cleaning) was carried out, under the close supervision of the Spanish Statistical Office, by the company awarded the contract.

All participants signed an informed consent form before responding to the survey questions. This research was conducted in accordance with the Declaration of Helsinki of the World Medical Association and with the regulation of European Union. The file data for public use does not require the approval of an accredited ethics committee for statistical or research purposes.

### Depression and Chronic Anxiety

Depression and chronic anxiety were assessed with the following yes–no questions: (1) “Have you ever been diagnosed with depression?”; (2) “Have you ever been diagnosed with chronic anxiety?”. It was considered that the participants that responded yes to the questions had suffered from depression/chronic anxiety. The question for depression has been previously validated (18), and the question for anxiety has been widely used in previous scientific literature (19–23).

### Diabetes and Cataracts

Diabetes and cataracts were assessed with the following yes–no questions: (1) “Have you ever been diagnosed with diabetes?”; (2) “Have you ever been diagnosed with cataracts?”. It was considered that the participants that responded yes to the questions had suffered from diabetes/cataracts. Self-reported diabetes is a valid and reliable measure (24–32). Self-report accuracy is also high for cataract (33). Self-report questionnaires are widely used in large population studies where it is difficult to collect clinical data, and the questions used in this study to assess diabetes and cataracts have been already used in various nation-wide studies (20–23, 34).

### Covariates

The selection of the control variables was based on bivariate analyses (Table 1) and on past literature (34–37). Sociodemographic variables included gender, age, marital status, living as a couple and education. Education was based on the highest educational level achieved and was categorized as ≤ primary, secondary, and ≥tertiary. Marital status was categorized as married and single/widowed/divorced/separated. Living as a couple was categorized as yes/no. Smoking status was self-reported and categorized as never, current smoker, and past smoker. Alcohol consumption in the last 12 months was self-reported and categorized as yes (any) and no (none). Height and weight were self-reported. Body mass index (BMI) was calculated as weight in kilograms divided by height in meters squared. Obesity was defined as BMI ≥ 30 kg/m^2^, according to World Health Organization (https://www.who.int/health-topics/obesity).

**Table 1:** Prevalence of depression and chronic anxiety in Spanish adults with diabetes (overall, by cataracts status and by covariates).

**Variables**	**Categories**	**Depression**	**Chronic anxiety**
Overall	- (*n* = 2,266, 100%)	417 (18.4)	279 (12.3)
Cataracts^a^	Yes (*n* = 768; 33.9%)	190 (24.7)	108 (14.1)
	No (*n* = 1,498; 66.1%)	227 (15.2)	171 (11.4)
Gender^a^, ^b^	Women (*n* = 1,138; 50.2%)	301 (26.4)	195 (17.1)
	Men (*n* = 1,128; 49.8%)	116 (10.3)	84 (7.4)
Age^b^	≥65 years (*n* = 1,564; 69.0%)	301 (19.2)	178 (11.4)
	<65 years (*n* = 702; 31.0%)	116 (16.5)	101 (14.4)
Marital status^a^, ^b^	Married (*n* = 1,266; 55.9%)	187 (14.8)	128 (10.1)
	Single/widowed/divorced/separated (*n* = 997; 44.1%)	230 (23.1)	151 (15.1)
	Missing (*n* = 3)	-	-
Living as a couple^a^, ^b^	No (*n* = 1,004; 44.4%)	232 (23.1)	148 (14.7)
	Yes (*n* = 1,255; 55.6%)	184 (14.7)	131 (10.4)
	Missing (*n* = 7)	-	-
Education^a^	≤ Primary (*n* = 1,398; 61.7%)	287 (20.5)	168 (12.0)
	Secondary (*n* = 638; 28.2%)	99 (15.5)	89 (13.9)
	≥Tertiary (*n* = 230; 10.1%)	31 (13.5)	22 (9.6)
Smoking^a^, ^b^	Never (*n* = 1,153; 50.9%)	246 (21.3)	151 (13.1)
	Past (*n* = 777; 34.3%)	104 (13.4)	74 (9.5)
	Current (*n* = 335; 14.8%)	67 (20.0)	54 (16.1)
	Missing (*n* = 1)	-	-
Alcohol^a^, ^b^	No (*n* = 1,179; 52.1%)	277 (23.5)	181 (15.4)
	Yes (*n* = 1,085; 47.9%)	140 (12.9)	97 (8.9)
	Missing (*n* = 2)	-	-
Obesity	No (*n* = 1,399; 66.5%)	238 (17.0)	160 (11.4)
	Yes (*n* = 704; 33.5%)	144 (20.5)	98 (13.9)
	Missing (*n* = 163)	-	-
Physical activity^a^, ^b^	<600 MET-min/week (*n* = 359; 35.4%)	81 (22.6)	62 (17.3)
	≥600 MET-min/week (*n* = 655; 64.6%)	89 (13.6)	75 (11.5)
	Missing (*n* = 1,252)	-	-

*Values expressed in Frequencies (Valid %).*

*Significant differences between groups were calculated with chi-square tests.*

a *Significant differences in the prevalence of depression.*

b *Significant differences in the prevalence of chronic anxiety.*

*Those covariates that were significant were included in the regression models (Tables 2, 3)*.

The International Physical Activity Questionnaire (IPAQ) short form was used to measure physical activity. The unit of physical activity used was MET-minutes/week, where MET is the Metabolic Equivalent of Task. Total physical activity MET-minutes/week were calculated through the following formula: *sum of Walking* + *Moderate* + *Vigorous MET-minutes/week scores* (38). Participants were divided in two categories according to the guidelines for data processing and analysis of the IPAQ (38) and according to the American Diabetes Association PA guidelines (39): (1) fewer than 600 MET-min/week and (2) at least 600 MET-min/week, equivalent to meeting current PA recommendations. IPAQ was developed for population surveillance of physical activity among adults aged 15–69 years, and its use with older and younger age groups is not recommended (38). Therefore, IPAQ was completed only by people aged 15-69 years and, to avoid losing all the data of people ≥ 70 years, a missing category for physical activity was included in the regression analyses. IPAQ has been validated in adult populations from different countries showing acceptable validity (ρ = 0.30, 95% CI: 0.23-0.36) and reliability (Spearman's ρ = 0.81, 95% CI: 0.79-0.82) (40). Specifically, IPAQ Short Form has been validated among Spanish university students showing adequate validity (41).

### Statistical Analysis

The statistical analysis was performed with SPSS 23.0 (IBM, NY, USA). The prevalence of self -reported depression and chronic anxiety in Spanish adults with diabetes was studied, analyzing the differences by cataracts status and by covariates (Table 1). These differences were assessed by Chi-squared tests and those covariates that were significant were included in the regression models (Tables 2, 3).

**Table 2:** Associations between cataracts (exposure) and covariates with depression (outcome) in Spanish adults with diabetes, estimated by multivariable logistic regression (overall and by gender).

	**Overall (*n* = 2,266)**	**Men (*n* = 1,128)**	**Women (*n* = 1,138)**
Cataracts (REF no cataracts)	1.655 (1.295–2.115)^***^	1.480 (0.954–2.296)	1.762 (1.307–2.374)^***^
Female gender (REF male gender)	3.005 (2.252–4.010)^***^	–	–
Single/widowed/divorced/separated (REF married)	1.064 (0.524–2.161)	1.517 (0.513–4.489)	0.809 (0.324–2.022)
Not living as a couple (REF living as a couple)	1.389 (0.682–2.827)	2.201 (0.751–6.445)	0.977 (0.388–2.456)
Primary Education (REF Tertiary education)	1.098 (0.711–1.695)	1.571 (0.797–3.094)	0.787 (0.435–1.422)
Secondary Education (REF Tertiary education)	0.978 (0.622–1.537)	1.219 (0.598–2.484)	0.762 (0.413–1.406)
Past smoking (REF never)	0.719 (0.526–0.983)^*^	0.752 (0.452–1.251)	0.746 (0.488–1.140)
Current smoking (REF never)	0.513 (0.355–0.742)^***^	0.565 (0.310–1.030)	0.498 (0.306–0.809)^**^
Alcohol (REF no)	0.671 (0.522–0.862)^**^	0.557 (0.374–0.830)^**^	0.748 (0.544–1.028)
Physical activity <600 MET-min/week (REF ≥ 600)	1.686 (1.192–2.385)^**^	1.439 (0.828–2.500)	1.865 (1.191–2.922)^**^

*REF, Reference Category.*

*Values expressed in Odds Ratio (95% Confidence Interval).*

* *P < 0.05.*

** *P < 0.01.*

*** *P < 0.001.*

*Model adjusted for gender (except the gender-stratified analyses), marital status, living as a couple, education, smoking, alcohol, physical activity*.

**Table 3:** Associations between cataracts (exposure) and covariates with chronic anxiety (outcome) in Spanish adults with diabetes, estimated by multivariable logistic regression (overall and by gender).

	**Overall (*n* = 2,266)**	**Men (*n* = 1,128)**	**Women (*n* = 1,138)**
Cataracts (REF no cataracts)	1.343 (0.999–1.805)	1.014 (0.568–1.811)	1.519 (1.067–2.163)^*^
Female gender (REF male gender)	2.699 (1.944–3.745)^***^	–	–
≥65 years (REF <65 years)	0.756 (0.492–1.160)	0.298 (0.123–0.727)^**^	1.125 (0.659–1.920)
Single/widowed/divorced/separated (REF married)	1.890 (0.846–4.224)	0.769 (0.213–2.784)	3.727 (1.240–11.205)^*^
Not living as a couple (REF living as a couple)	1.467 (0.653–3.298)	0.459 (0.128–1.652)	3.418 (1.132–10.321)^*^
Past smoking (REF never)	1.371 (0.958–1.961)	2.071 (1.039–4.131)^*^	1.220 (0.750–1.984)
Current smoking (REF never)	1.971 (1.308–2.970)^**^	2.979 (1.403–6.326)^**^	1.664 (0.962–2.880)
Alcohol (REF no)	0.644 (0.482–0.861)^**^	0.404 (0.254–0.642)^***^	0.868 (0.605–1.246)
Physical activity <600 MET-min/week (REF ≥ 600)	1.478 (1.015–2.151)^*^	1.025 (0.557–1.889)	1.925 (1.179–3.144)^**^

*REF, Reference Category.*

*Values expressed in Odds Ratio (95% Confidence Interval).*

* *P < 0.05.*

** *P < 0.01.*

*** *P < 0.001.*

*Model adjusted for gender (except the gender-stratified analyses), age, marital status, living as a couple, smoking, alcohol, physical activity*.

Multivariable logistic regression examined association between cataracts (exposure) and depression or anxiety (mental health outcome) in men and women. Goodness-of-fit and diagnostic tests were conducted in order to check the suitability of the regression model. The model for depression was adjusted for gender (except the gender-stratified analyses), marital status, living as a couple, education, smoking, alcohol, physical activity. The model for chronic anxiety was adjusted for gender (except the gender-stratified analyses), age, marital status, living as a couple, smoking, alcohol, physical activity. All variables were included in the models as categorical variables. Results from the logistic regression analyses are presented as odds ratios (ORs) with 95% confidence intervals (CIs). There were some missing data for the following variables: marital status (*n* = 3: 0.13%), living as a couple (*n* = 7: 0.31%), smoking (*n* = 1: 0.04%), alcohol (*n* = 2: 0.09%), obesity (*n* = 163: 7.19%) and physical activity (*n* = 1,252: 55.25%). The level of statistical significance was set at *p* <0.05.

## Results

In this self-weighting sample of 2,266 adults with diabetes residing in Spain (50.2% women; average age 69.7 ± 12.7 years, males 67.9 ± 11.9 and females 71.4 ± 13.2; age range 15-98 years, males 15-97 and females 26-98), the overall prevalence of depression was 18.4% and the prevalence of chronic anxiety was 12.3 %. The prevalence of depression among those with cataract was significantly higher than in those without cataract (24.7 vs. 15.2%). Bivariate analysis showed that the groups with the following characteristics had the highest levels of depression: women, ≥65 years, people not married, people not living as a couple, ≤ primary education, people who never smoked, non-alcohol drinkers, obesity, <600 MET-min/week of physical activity. For chronic anxiety groups the following characteristics had the highest levels of chronic anxiety: women, <65 years, not married or not living as a couple, secondary education, current smokers, non-alcohol drinkers, obesity, <600 MET-min/week of physical activity (Table 1).

Multivariable logistic regression showed that, overall, the presence of cataract was associated with significantly higher odds for depression (OR = 1.655; 95% CI = 1.295-2.115). In the gender-stratified analyses, cataracts were significantly associated, only in women, with higher odds for depression (OR = 1.762; 95% CI = 1.307-2.374) and for chronic anxiety (OR = 1.519; 95% CI = 1.067-2.163) (Tables 2, 3).

The suitability of the regression models was assessed. The final model for predicting depression was a significant improvement in fit over the null model [χ^2^_(14)_ = 160.016, *p* < 0.001], Pearson's chi-square test indicated that the model fitted the data well [χ^2^_(353)_ = 350.802, *p* = 0.523] and the deviance chi-square indicated a good fit [χ^2^_(353)_ = 378.894, *p* = 0.164]. The model for predicting chronic anxiety showed a significant improvement in fit over the null model [χ^2^_(13)_ = 99.045, *p* < 0.001], Pearson's chi-square test indicated that the model fitted the data well [χ^2^_(235)_ = 235.049, *p* = 0.487] and the deviance chi-square indicated good fit [χ^2^_(235)_ = 251.149, *p* = 0.224].

## Discussion

In this large representative sample of Spanish adults with diabetes, we find, for the first time, that women suffering from diabetes and cataracts show significantly higher odds for depression and for chronic anxiety compared to those women who had diabetes but no cataracts.

While the present findings support previous studies showing higher levels of depression and chronic anxiety in people with diabetes (7, 42), it adds to literature by demonstrating that women who have both diabetes and cataract are at much greater risk of mental health complications compared to women with diabetes but no cataract. Also, women who have both diabetes and cataract are at greater risk of mental health complications compared to men with the same conditions, as the association between cataract and mental health was not significant in men with diabetes. The increased risk of depression and chronic anxiety in women with diabetes who have cataracts is of concern as this dual impairment is likely to continue to increase owing to an aging population which leads to both increased prevalence of diabetes and cataracts (43, 44).

There are several plausible mechanisms that may explain the higher risk of depression in people with diabetes who also have cataracts. First, having more than one condition will amplify depression, as multiple conditions require more adjustments for activities of daily living and managing diabetes in the presence of blurred vision (caused by cataracts). Second, managing diabetes and cataracts may need additional medical interventions, and literature suggests that medical intervention is linked to discomfort and uncertainty (45) and is a stressor for depression. Third, if depression is managed through anti-depression medication, this may actually worsen the status-quo as these have been shown to be a risk factor for cataracts (46). Fourth, people with diabetes who also have cataracts experience reduced vision-related changes in quality of life, which might contribute to the development of depression (47).

Possible reasons for cataracts to be significantly associated with higher odds for depression and anxiety in women with diabetes but not in men could be explained by some biological and genetic differences between men and women. Recent evidence suggests that biological factors such variations in ovarian hormone levels and particularly decreases in estrogen may contribute to the increased prevalence of depression and anxiety in women (48). Women with cataracts are more affected than men with regards to the cataract-related medical interventions that may be required (45) and about the vision-related changes in the Quality of Life caused by cataract (47). Previous research suggests that women also have a lower tolerance for stress than men (49). Future studies should explore these reasons in detail.

The large representative sample of the Spanish population and the novel associations investigated are clear strengths of this study. Findings, however, must be interpreted in light of the study limitations. First, the study is cross-sectional in nature thus it is not known whether those with diabetes and anxiety or depression develop cataracts or those with diabetes and cataracts develop anxiety or depression. It is likely bidirectional. A longitudinal study would further explore the identified associations. Second, participants were asked “have you ever been diagnosed with cataracts,” “have you ever been diagnosed with anxiety (or depression),” and “have you ever been diagnosed with diabetes.” It is therefore possible that those participants who reported affirmatively to all questions did not have all conditions at the same time. Also, it is possible that people who responded affirmatively to having cataracts might have had their cataract removed. If that was the case, then we should in fact see higher odds ratios in people who have not had the operation, and it is possible that what we have shown may be underestimate. Also, all questions were self-reported, introducing self-reporting and recall bias into the findings. Finally, there was information about the chronic conditions studied in the present study (diabetes, cataract, depression and chronic anxiety) that were not included in the survey, such as duration of the disease, treatment and type or severity, being recommendable that future studies consider also these aspects.

## Conclusions

In conclusion, cataracts are a significant risk factor for depression and chronic anxiety in Spanish women with diabetes, but not in men. It would be important for healthcare practitioners to take this into account, and manage both cataract and diabetes with appropriate and timely interventions to reduce the risk of further mental health complications, especially in women.

## Data Availability Statement

The raw data supporting the conclusions of this article will be made available by the authors, without undue reservation.

## Ethics Statement

Ethical review and approval was not required for the study on human participants in accordance with the local legislation and institutional requirements. Written informed consent to participate in this study was provided by the participants' legal guardian/next of kin.

## Author Contributions

All authors listed have made a substantial, direct, and intellectual contribution to the work and approved it for publication.

## Conflict of Interest

The authors declare that the research was conducted in the absence of any commercial or financial relationships that could be construed as a potential conflict of interest.

## Publisher's Note

All claims expressed in this article are solely those of the authors and do not necessarily represent those of their affiliated organizations, or those of the publisher, the editors and the reviewers. Any product that may be evaluated in this article, or claim that may be made by its manufacturer, is not guaranteed or endorsed by the publisher.
